# Effects of 6 Weeks Direct Instruction and Teaching Games for Understanding Programs on Physical Activity and Tactical Behaviour in U-12 Soccer Players

**DOI:** 10.3390/ijerph17145008

**Published:** 2020-07-12

**Authors:** Juan Vicente Sierra-Ríos, Filipe Manuel Clemente, Ezequiel Rey, Sixto González-Víllora

**Affiliations:** 1 Physical Education Department, Faculty of Education, University of Castilla-La Mancha, 16071 Cuenca, Spain; juanvisr@gmail.com; 2 Escola Superior Desporto e Lazer, Instituto Politécnico de Viana do Castelo, Rua Escola Industrial e Comercial de Nun’Álvares, 4900-347 Viana do Castelo, Portugal; filipe.clemente5@gmail.com; 3 Instituto de Telecomunicações, Delegação da Covilhã, 1049-001 Lisboa, Portugal; 4 Faculty of Education and Sport Sciences, University of Vigo, 36005 Pontevedra, Spain; zequirey@uvigo.es

**Keywords:** association soccer, pedagogical models, accelerometry, youth sport, game performance, attack tactical principles, soccer

## Abstract

The purpose of this study was to compare the effects of 6 weeks direct instruction and teaching games for understanding (TGfU) programs on the decision-making and execution (post-interventions), as well, as on the physical activity (PA) levels during sessions. Thirty under-12 football players participated in this study (age: 10.3 ± 0.45 years) and were randomly assigned to TGfU (*n* = 15) or direct instruction (*n* = 15) group. Two sessions/week were implemented. Results revealed that TGfU promoted higher levels (*p* = 0.043; d = 2.99) of light PA (28.96%) compared with direct instruction (27.55%). Non-significant higher sedentary PA levels (*p* = 0.073; d = 2.62) were found in the control group (35.48%). In terms of tactical principles, conservation of the ball increased the percentage of moderate to vigorous physical activity in TGfU (43.60%) compared with direct instruction (38.05%). According to the Game Performance Evaluation Tool (GPET), significant improvements (*p* = 0.018, d = 3.78) of the attacking player with the ball in the percentage of change between groups in the unsuccessful execution in TGfU (% = −62.2) were observed compared with direct instruction (% = 14.2). TGfU seems to be more appropriate than direct instruction to increase the light PA levels during sessions while no significant differences were found between programs in moderate and vigorous intensities. Regarding the effects of programs in decisions, greater improvements in decisions with the ball were found in TGFU compared to DI.

## 1. Introduction

The contextual nature of soccer presents a high degree of uncertainty. Thus, game dynamical properties are necessary to develop different skills, which allow the player to adapt to situations that arise during the game, aiming to improve the performance [1]. 

To promote more integrative learning, cognitive psychology has proposed alternative teaching methodologies based on an ecological point of view of teaching in sports, being one of the most common approaches to teaching games for understanding (TGfU) [2]. The TGfU model is a contextualized teaching approach based on the use of modified games (MG), to enhance the motivation and decision-making of athletes, favouring the transfer of learning to other sports [3]. Moreover, the TGfU model is an alternative to direct instruction of sports teaching proposing innovation in the teaching−learning process and prioritization of contents towards a modification of the structuring of functional and formal elements [4].

Direct instruction (DI) is aimed at the ability to execute a technical approach to teaching game skills (technical components) such as control, passing, or driving [4], with sessions in which the repetition of the technique to learn was the aspect to which most of the time was devoted [5], with greater control of the coach in the teaching−learning process, where the players follow his instructions [6] and improve the coordinating ball skills.

The direct instruction due to the automation of the mechanical movements carried out causes that the actions they execute are not understood by the students, so they depend on the instructions of their coach [7]. Sports education from a traditional approach generates some drawbacks, among which monotony and standardization stand out, as well as lack of motivation or the little time dedicated to promoting decision making [5], limiting creative development by using direct instruction methodology.

Modified games (MG) are common in these models trying to develop tactical awareness throughout an emphasis on exploration and problem solving [8]. Additionally, modified games are also used aiming to increase intensity while playing, thus possibly favoring the development of some fitness variables, such as cardiorespiratory ones [9]. As a multidimensional task, modified games also allow to influence the frequency and type of technical actions and skills [10], tactical and collective behavior [11], and decision-making aspects [12]. For those reasons, some researchers discuss the importance of models that promote tactical skills whose results show significant improvements in decision making [13]. This aspect can be due to the increased difficulty of the tactical components via modification of the elements of the game, such as the size of the pitch or the number of players. Within this scope, the levels of learning in U-8, U-10, and U-12 soccer players were compared, with improvements in declarative and procedural knowledge, as well as technical-tactical skills in both the tactical principles of attack as in defense [14].

Previous studies [15] have shown that the teaching of sport through tactical models allows players to accumulate more than 50% of the time in moderate to vigorous physical activity (MVPA) in sports, such as in rugby (52.9%) or soccer (57.9%), by measuring with pedometers. Moreover, [16] indicated that comparing traditional and comprehensive methodologies, players in the comprehensive group accumulated significantly more time in MVPA than the DI. Moreover, comprehensive methodologies based on modified games and with greater possibilities of contact with the ball, allow an increase of vigorous physical activity (VPA) opportunities [17] and a maximum heart rate of around 90–95% [18]. Thus, MG reveals improvements at a physical, technical-tactical, and physiological level [19]. In modified games (e.g., 2 × 2 or 3 × 3), participation actions are greater, being able to relate to the ability to perceive tactical contents and consider them as an evolution in pedagogical complexity [20].

Although TGfU’s primarily objective is to improve students’ understanding of games and develop tactical knowledge, another important factor is how this model promotes physical activity (PA) levels. In this respect, [21] measured the levels of PA in a soccer didactic unit in a school environment by using small-sided games, promoting the accumulation of 50% of the time of the session in moderate to vigorous physical activity (MVPA). Moreover, [22] researched the physical activity levels in five training sessions using both approaches with accelerometers and finding similar levels of physical activity. In the case of the youth sports training sports, there are few studies [16,21] comparing the effects of TGfU and the traditional teaching approach in both physical activity levels and decision making using a game-centered approach soccer unit in physical education classes with male and female young students, establishing higher levels of physical activity in models that prioritize tactical aspects compared to direct instruction. 

Due to the little research that exists on this research topic [16,21], it would be important to increase the understanding about how these game-based models may lead to changes in both PA levels and decision making compared to more traditional approaches based on direct instruction (DI). That may allow us to understand the magnitude of effects of these programs and identify the main variables that may benefit from game-related models. Thus, this study aims to understand the effects of structured TGfU and DI teaching programs on decision making and execution, as well as to analyze the effects on PA levels during the session in under-12 soccer players. The knowledge about the real effects will help to identify the most adequate strategies to promote greater PA levels within the classes and which kind of models may also be more beneficial to understand the dynamics of the game and the decision making. 

## 2. Materials and Methods 

### 2.1. Experimental Approach to the Problem

This study followed a parallel study design. Thirty players were randomly assigned to two intervention groups, one consisting in TGfU (*n* = 15) and one in DI (*n* = 15). Both experimental groups had two intervention sessions a week for a period of six weeks. Baseline and post-intervention analysis were conducted of decision-making and execution while playing a specifically designed game. The experimental approach occurred from January to March with two groups of U-12. As a result, 12 experimental sessions were employed with a mean duration of 80 min each. In all training sessions, PA levels were monitored using accelerometers. 

### 2.2. Participants

The present study used a non-probabilistic inter-subject case design for the convenience of two U-12 teams. Table 1 shows the information on the age, height, weight, and body mass index of each team. Sample size calculation was made for an alpha of 0.05 and a power of 0.8. The results indicated a recommended sample of 28 or more measurements/surveys are needed to have a confidence level of 95% for the real value to be within ±5% of the measured/surveyed value. Thirty male players belonging to the same club and having 6.9 ± 1.08 years of training experience participated in the study (one team in the experimental group, who underwent the TGfU, and the other team in the control group, who experienced the DI). The inclusion criteria were: (i) the players had previous experience with the DI, but not with the TGFU; and (ii) the players had a minimum experience in soccer of 6.9 ± 1.08 years. The players regularly performed two weekly training sessions (~80 min per session). The team also regularly competed for one official match per week. The current study described 12 training sessions over 6 weeks. The study was developed after 9 weeks at the beginning of the season. The players had the same competitive and playing skill level. Players were randomly assigned to the groups, and all the participants were present in all training sessions using an accelerometer. They had to belong to the team and have done both the pre-test and post-test in the Game Performance Evaluation Tool (GPET). According to the high quality, the study protocol was approved by the scientific committee. The research followed every ethical standard throughout including data collection and data analysis. Authors/researchers paid extraordinary attention to those requirements related to privacy, confidentiality, and informed consent. Besides, players to be included in the research gave informed consent under the Declaration of Helsinki indicating that their voluntary participation which was obtained from the players and their legal representatives after the explanation of the experimental protocol and the possible benefits and risks of the research project. Finally, these requirements were adapted to a social sciences and educational context. 

### 2.3. Anthropometry

Anthropometry measurement was made according to Kushner’s protocol [23]. The body mass and height were measured twice with a five-minute interval between measurements. The body mass was measured at the nearest 100 g using a calibrated digital scale (SECA Model 861, Vogel and Halke, Hamburg, Germany) with slightly dressed children without shoes. The height was measured to the nearest millimeter using a wall-mounted stadiometer, with children standing directly against the wall without shoes, to align the spine with the stadiometer. The head was placed so that the chin is parallel to the floor. The mean of the two measures of body mass and height was used to calculate the BMI as the body mass in kilograms divided by the square of the height in meters (kg/m^2^). 

### 2.4. Physical Activity

The wGT3X accelerometers (three axes, 100 Hz, Actigraph) were used to measure the PA levels during training sessions. The instrument was previously tested for its validity and reliability to measure PA levels [24]. The device was placed on the right hip, above the iliac crest, near the center of gravity [25]. The accelerometers were programmed at 100 Hz and exported in epoch of 1 s. The cut points obtained from [26] for the accelerometers were sedentary PA, 0–100 counts per minute (CPM); light PA, 100–2295 CPM; moderate PA, 2296–4012 CPM; and vigorous PA, ≥ 4013 CPM. The data was analyzed using the Actilife 6.0 software.

### 2.5. Game Performance Evaluation Tool (GPET)

This instrument evaluates the game’s performance from a tactical perspective, in which decisions and execution are analyzed and codified [27]. The player is evaluated according to the decisions he makes or executes. Decision making is evaluated for every decision you make and is analyzed on every move you make; this is called a decision-making unit (DMU). The components and tactical principles of attack were analyzed; keep the ball, progress to the opponent’s goal, and achieve a goal for the attacking player with the ball (AonB) and the attacking player without the ball (AofB). The components that were analyzed in the AonB were the execution of the control of the ball; the decision making of pass, driving, and shooting; and its success of execution, while the AofB measures the decision in the unmarks, supports, and movements without the ball and the success of the execution. These variables were coded with 1 (appropriate/success) or 0 (not appropriate/unsuccessful) according to the items of each of the existing variables. Each move made by the player, both AonB and AofB were analyzed by the decision-making unit. To analyze the reliability of the variables, Cronbach’s Alpha was analyzed (α = 0.75); the result obtained was superior to that indicated for the reliability of the statistical analyses (α = 0.70).

### 2.6. Intervention Programs

Two sports coaches with UEFA-B soccer qualification implemented both teaching approaches (TGfU and DI). The coach who conducted the sessions under the comprehensive methodology (TGfU) received prior training in agreement with [28], during two sessions of 80 minutes each. The main researcher gave the training lesson. The sessions were designed by him and supervised by experts in the model.

The players who participated in the direct instruction group developed the training using the analytical technique used so far, following the structure of (a) warm-up with continuous running and joint mobility; (b) main part with technical and analytical exercises according to the objective and a final time of 10 min in which they made a small game; and (c) to conclude with stretching exercises. 

On the other hand, the players that participated in the TGfU group made the practice based on small-sided games. These sessions had the following structure: (a) warm-up with a playful game with the ball; (b) an initial reflection was introduced in which the objective of the session was exposed; (b) the modified games were carried out with which, practically and experientially, the learning that arises was favored.

After having carried out the modified game, tactical awareness was carried out with an approximate duration of 5 min; in this phase, the coach asked questions within the aim of the session so that the players try to solve the problems that could arise. The coach offered positive feedback. Subsequently, with the answers related to the tactical aim of the session, a technical skill exercise was carried out as a corrective task, to improve the previous modified game and the assimilation of concepts.

Finally, the training concluded with a final reflection in which the aspects learned were transferred.

Table 2 shows the objectives of the sessions in the comprehensive model and the direct instruction model implemented in the two groups (TGfU and DI) based on Bayer [29].

### 2.7. Data Analysis 

The data were analyzed with the statistical program SPSS version 24.0 (Chicago, IL, USA). To check the normality of the variables, the Shapiro–Wilk test was used, since the sample was less than 50. Since the variables presented a normal distribution (*p* > 0.05) and homogeneity in the Levene test (*p* > 0.05), parametric tests were performed. The PA levels were adjusted by Body Mass Index (BMI) as a covariate to control for possible effects on physical activity levels. Both PA and GPET were compared using multivariate analysis (MANCOVA). Additionally, Cohens’ d was used to calculate effect sizes [30]. The magnitude of changes was classified based on the following thresholds: trivial (d < 0.2), small (0.2 ≤ d < 0.5), medium (0.5 ≤ d < 0.8), or large (d ≥ 0.8). Bonferroni was used to control the type I error. The level of statistical significance was set at *p* ≤ 0.05, with a confidence interval of 95%.

## 3. Results

MANCOVA results examining each type of PA between both approaches and the variables of decision making and execution of the GPET are presented in Table 3, Table 4, Table 5 and Table 6.

Table 3 shows the mean results of the PA levels in each of the groups (TGfU and DI) monitored over the experimental sessions. The players in the DI group presented higher levels of sedentary PA percentage. The players in the TGfU presented percentages of light PA significantly higher than the DI. Regarding the MVPA, no significant differences were found between the two models.

Analyzing the PA levels according to the Bayer attack principles [29], in Table 4, the levels of physical activity of the tactical principle of keeping possession of the ball, the percentages of sedentary physical activity were higher for DI to TGfU (36.55 and 26.28 respectively). For light and moderate PA, the TGfU presents differences with 30.11% and 14.35% against the DI with 25.38% and 11.37%. Finally, the percentage of MVPA is significantly higher in the TGfU (43.60%) than the DI (38.05%).

The tactical principle of progression had no significant differences between TGfU and DI. Finally, corresponding to the tactical principle of achievement of the objective, we also do not highlight significant differences between both models.

Concerning the tactical evaluation instrument GPET, no significant differences were found in the AonB in the decision making for the driving, passing, and shooting variables. However, significant improvements were made in the errors of the execution of the AonB. The players of the TGfU reduced the errors in the execution of the pass, driving, and shooting in proportion to the DI. Analyzing the percentage of change of AonB within each group, we can highlight a large increase in the success of the execution of the TGfU (42.04%) compared with the DI (12.5%) and a decrease of unsuccessful executions by the TGfU compared with the DI (−62.6% and 12.4%). These results can be seen in Table 5.

In Table 6, TGfU shows significant improvements in the decision making in the support variable and uncheck in the AofB with a mean of 7.30 in the pretest and of 9.00 in the post-test, thus reducing the errors in the decision making of the same variables 0.90 and 0.25 between the previous phase and the final phase. Besides, we can observe superior improvements in the execution success with 6.50 in the pretest results and 8.87 in the post-test and a decrease in execution errors with averages of 1.20 in the pre-test to 0.37 in the post-test, opposite to DI. Regarding the percentage of change within the group, the TGfU shows an improvement in decision making and a reduction in execution unsuccessful passes (23.3% and −72.2%) compared to DI (−14.3% and 32.72%). Similarly, there is an improvement in the execution of the uncheck and a decrease in errors in the TGfU (36.61% and −68.3%) compared with the DI (−9.90 and 23.0%).

## 4. Discussion

The main objective of the study was to compare the PA levels in extracurricular environment in U-12 soccerplayers using the accelerometer, as well as to analyze the decision making and execution after the application of two teaching models (DI vs. TGfU). According to the initial hypotheses, TGfU shows significantly higher levels of light physical activity than DI. However, no meaningful differences were found in determinant levels (moderate and vigorous PA). According to the tactical principles, keeping possession of the ball presents significantly higher levels of physical activity in the TGfU compared with the DI, less the sedentary PA that is higher in the direct instruction model. Concerning the second hypothesis, for decision-making, the AonB presents improvements in the TGfU, but it is not significant, while the AofB presents improvements related to DI both in decision-making and in execution.

### 4.1. Physical Activity

The investigation shows significant light PA levels in the TGfU compared to the DI, and the physical activity levels moderate, vigorous, and MVPA were higher in the TGfU than the DI, but they are not significant. The only variable in which the DI is superior to the TGfU, although not significantly, is sedentary physical activity; in the direct instruction, the players are more inactive.

While the levels of physical activity in the training sessions are not significant between pedagogical approaches, analyzing the training sessions according to the tactical principles between the TGfU and DI, the principle of keeping the ball using the TGfU is the most significant for developing MVPA and less sedentary PA. In the other tactical principles, penetrating the defense and attacking the goal, the DI had higher levels of physical activity, but they are not significant.

The results of the present study do not comply with the recommendations proposed by international institutions to overcome 50% of the time in MVPA [31], since the TGfU and DI players accumulated 37% of the time in MVPA. Despite this, in this study, the percentage of sedentary PA was lower in the TGfU than in the DI. 

Comparing MVPA levels based of previous studies, our research presents an improvement in MVPA levels compared to [32], who measured physical activity among 6–12-year-old children during soccer practice in one hour with 23% of MVPA, and [33], with a soccer training of 50 min, obtained 33%. On the other hand, [34] obtained better results of MVPA than our research. These studies present an average between 49% and 54% of MVPA during one hour of training, respectively. These results improve our study, since our research has a sports practice time of 80 min. Finally, we found MVPA levels comparable to our study in [35] with 36.8% or less than 50% of MVPA in soccer [36].

Analyzing the levels of physical activity with the application of pedagogical approach, [21] obtained significant differences in the percentage of MVPA in the comparison of directed instruction approaches with tactical methodologies (47.08% vs. 31.89%) in hockey, using the triaxial accelerometry RT3 and the System for Observing Fitness Instruction Time (SOFIT) [37]. These results are higher because the players had previous experience in tactical methodologies in contrast to our sample, since the participants were new to training using the TGfU methodology, during which, while assimilating the learning, physical activity is less. After all, they are standing longer. As the sessions progressed, this downtime was reduced. Nevertheless, [16] used the same instruments as Harvey in sports such as netball, rugby, and soccer, obtaining higher levels than this study in moderate to vigorous physical activity (50%). With other physical activity assessment instruments such as SOFIT, the group with tactical methodology presented better levels of MVPA compared to the traditional group. However, for the comparison of the studies presented, the use of different instruments to measure it should be considered.

In the present study, modified games based on the TGfU were designed in 3 vs. 3 and 4 vs. 4 format, with field measurements similar to those of [38] (30 × 25 m); the players were able to perform more movements at high intensity than at medium or low intensity compared to the group with DI training, highlighting the sessions in which the tactical principles of keeping the possession of the ball with an MVPA of 43.60% against 38.05%, respectively, were developed. These types of designs through modified games allow greater possibilities of contact with the ball, with a significantly greater increase in MVPA in 3 vs. 3 formats with 11.64 min [39]. The modified games present improvements at a physical, technical−tactical, and physiological level [19]. Nevertheless, to obtain some levels of significant MVPAs, we have to consider a progression and evolution of tactical complexity [20] with participation in small groups (for example, 2 × 2 or 3 × 3) where the actions of the players in contact with the ball are more constant. Our training sessions were designed in reduced 3 × 3 or 3 × 3 games with a joker and varying ball touches allowing players to constantly move increasing levels of physical activity.

The use of various types of accelerometers used in other studies, such as RT3 in [21] or wGT3X in our study, differs in showing superior results in tactical models compared with traditional ones. We can establish that accelerometers can be more sensitive to outpatient activities, such as walking and running, compared to specific activities, such as shooting, passing, or dribbling, and are potentially the most frequently performed movements in soccer. This is because the accelerometer reads movement from the area of the body where the device is placed instead of its parts [40]. We can especially consider in the first sessions that the time of placement of the accelerometers in the players was greater due to learning in the correct position so that they could effectively measure the levels of physical activity. As the sessions progressed, the placement of the accelerometer became faster.

Finally, as it is a novel methodology, more practice is needed by players and coaches to increase MVPA levels, since without previous experience, players require more time in tactical awareness for the assimilation of tactical components during the training. Longer tactical awareness time translates to longer sedentary PA time. Despite this, in this study, the percentage of sedentary PA was lower in the TGfU than in DI.

### 4.2. Game Performance Evaluation Tool (GPET)

The analysis of the tactical evaluation instrument (GPET) between both approaches can highlight in the TGfU in the AonB significant improvements in the execution success with a change percentage of 42.04% with 12.5% of the DI. However, we did find significant differences in the unsuccessful actions of TGfU with the DI in the execution on AonB with a percentage of change between the pre-intervention phase and the subsequent one of −62.6% and 12.4%, respectively. 

On the other hand, assessment the AofB, TGfU presents significant differences in the percentage of change in all the variables with the DI, both the success in the execution in decision-making (23.3% and −14.3% respectively) and decrease in unsuccessful of decision-making (−72.2% and 32.72). Besides, there is an improvement in the success of the execution of the uncheck in the TGfU compared with the DI (36.61% and −9.9%) and a reduction of unsuccessful executions of unchecks (−68.3% and 23.0%), respectively. This may be due to many movements the AofB have to make to find free spaces and be able to receive the pass from their partner.

In the decision-making process, we can establish in our study that the attacking player without the ball trained by TGfU performed an improvement in decision making in unmarking, as established by [14], who obtained similar results, in percentages, to the results in the present study on the effectiveness of decision making (78.1%) and execution (66.7%) with the AofB. Players at these ages increase the number of supports, [41] looking for the free spaces of a rival brand, to be able to receive the ball from their teammates and progress to attack the goal. Similar results were found in the support and fixing of variables in other studies [42]. Through TGfU training, players understand the effective way to perform unchecks, increasing the amount of unchecks and having the right decision making.

Players make more passes, reduce driving, and stop being so individualistic [28], giving greater importance to support, increasing their number and success, being an essential action in the evolution of the player [41]. Supports and unchecking are the skills that evolve the most as age increases and have greater importance in their evolution [41]. These actions are especially important because most of the time, the players hardly have contact with the ball [43].

Through the design of training sessions with the use of modified games in numerical superiority, we can increase decision making and the execution of unchecks in our research. We can affirm better results in decision making and pass execution, promoting the creation of free spaces by the AofB, less pressure to pass brand free companions, and carrying out a better execution, as well as improving support and uncheck movements [44].

At this time (U-12), the playing roles (defense, middle, and forward) are appreciated [43], highlighting the greater participation in those players with a higher level of experience, thus performing cooperative actions with their peers and promoting game performance [14,28]. By ages, [45] in U-10, the AonB presents a success in the executions of the 80%, as well as in U-14 (88%) [41]. For its part, the AonB also obtained similarity in decision making in driving, although no significant differences were found. In this study, this is reflected in the fact that the passes are shown backwards or that the field of play is given a greater amplitude, to keep the ball, change direction, or continue and deepen on the same side.

Measure up to pedagogical approaches, [44] obtained significant differences in favor of non-linear pedagogies versus management models throughout 14 sessions through games modified in the AonB in the pass and driving variables, unlike the results of the investigation. This may be due to the tactical awareness in which the players through the feedback are aware of the mistakes that the players make, improving decision making and execution to promote meaningful learning. In our research, players had more problems assimilating corrections when the player had the ball in them, and tactical awareness was longer so that players could assimilate the correct aspects.

Studies with other tactical analysis instruments [46] used TSAP [47] in U-14 children with a TGfU soccer teaching unit. The results suggest a strong interrelated process between video-based performance analysis and physical practice, with important comments being a tool to improve game performance in a short period. In our case, video-based performance analysis can be very effective to use during tactical awareness to check for errors that have occurred throughout the modified game and improve it later when the activity is repeated. Other instruments such as the FUT-SAT [48] compare the tactical behavior of soccer between U-12 and U-13, without finding significant differences in the offensive aspect [49].

Using the Game Performance Evaluation Tool in other sports, such as futsal, [50] investigated the performance of the game and differences in decision-making and execution of the AonB in the approval and management of soccer skills with TGfU, highlighting an improvement in pass decision making after the intervention in players with no prior experience in the approach, obtaining results similar to those of our research. This may be because these students developed more improved knowledge, leading to more tactically appropriate decisions [51]. In our case, the players also had no previous experience in the model and the application period of 6 weeks was too short to obtain more extensive results.

Other sports, such as basketball [52], compare TGfU and DI through the systematic observation of decision making and the real-life execution of the game, proposed by [53] and adapted from [54] in U-14 players. There are significant differences in decision making after the training program with a percentage change in the TGfU (42.8%) compared to the DI methodology (0%).

This study has some limitations; the sample, in which thirty players participated, is small. Moreover, the training of the coach and the players were accustomed to direct instruction and needed adaptation to consolidate the TGfU. Future studies should be expanded in the extracurricular field, with a larger sample with annual planning, also compared by gender, as there are more and more girls linked to soccer, and a comparison between the categories of the initiation stages (U-8 and U-10).

As practical implications, the results of this study allow us to highlight the importance of applying models such as TGfU, which encourages a reduction in sedentary activity. As well as an improvement in the tactical knowledge of AonB as AofB.

As teaching research and an innovation prospect, it would be interesting to expand this project in the extracurricular scope, with a larger sample, as well as to carry out annual planning, also comparing sports practice by gender, as there are more and more girls playing soccer. Moreover, a comparison could be made between the categories of formative soccer, highlighting especially the initiation stages (U-8 and U-10), to assimilate the learning and have them consolidated in stages of later formation (U-12, U-14, or U-16).

## 5. Conclusions

The research aims to compare two pedagogical approaches (TGfU and DI) analyzing the levels of PA, decision making, and execution. The TGfU is a good teaching approach to reduce the sedentary physical activity compared to the DI, both in the design of training sessions and the practical sessions designed and implemented according to the offensive tactical principles. We can also establish the improvements of the players who train through tactical models, since their decision making and execution are better, especially, when the attacking players without the ball perform unchecks and support adequately and optimally.

It is relevant to educate through tactical models based on practice, such as teaching game for understanding, to give importance to the player in their learning, that they can be the character of the game; the players empower better decision-making and execution. The development of the strategy in the game evolves the young player in his tactical intelligence.

## Figures and Tables

**Table 1:** Information the subjects about age, height, weight, and Body Mass Index (BMI) according to the pedagogical approach (Teaching Games for Understanding [TGfU] vs. Direct Instruction [DI]).

	TGfU (*n* = 15)	DI (*n* = 15)
	M (SD)	M (SD)
Age (years)	10.01 (0.07)	10.60 (0.57)
Height (m)	1.44 (0.06)	1.44 (0.07)
Weight (kg)	43.60 (9.17)	39.51 (6.48)
Body Mass Index BMI (kg/m^2^)	20.83 (3.47)	18.93 (2.07)

Note. *n*: sample; M: average; SD: standard deviation; m: meter: kg: kilograms.

**Table 2:** Training aims by training session and pedagogical approach.

Number of Training	TGfU	DI
1	Generate free spaces	Make passes
2	Generate free spaces	Make passes
3	Progression in numerical superiority in ball output	Create combined actions at the start of the ball
4	Progression in numerical superiority with passes	Create combined actions with passes
5	Progression in numerical superiority with driving	Dribbling
6	Progression in numerical superiority creating wide spaces	Uncheck support and break
7	Progression in numerical superiority creating wide spaces	Uncheck support and break
8	Progression in numerical superiority with passes	Create combined actions with passes
9	Progression in numerical superiority with oriented control	Performed control
10	Progression in driving 1 vs. 1	Dribbling 1 vs. 1
11	Progression in numerical superiority by bands	Create combined actions in bands
12	Finish from outside the area	Finish from outside the area

**Table 3:** Differences in physical activity levels according to the pedagogical approach (Teaching Games for Understanding [TGfU] vs. Direct Instruction [DI]).

		TGfU (*n* = 15)	DI (*n* = 15)	*p*	d
		M (SD)	M (SD)		
Sedentary PA	%	33.64 (0.72)	35.48 (0.68)	0.073	2.62
Light PA	%	28.96 (0.48)	27.55 (0.46)	0.043	2.99
Moderate PA	%	12.20 (0.30)	11.86 (0.28)	0.457	1.17
Vigorous PA	%	25.19 (0.49)	25.07 (0.27)	0.867	0.30
MVPA	%	37.39 (0.59)	36.95 (0.56)	0.606	0.76

Note. *n*: sample; %: percentage; M: average; SD: standard deviation; PA: physical activity; MVPA: moderate to vigorous physical activity; d: Cohen’s d.

**Table 4:** Physical activity level according to attacking principles.

PA Levels (%)	Models	Tactical Principle of Keeping Possession of the Ball			Tactical Principle of Penetrating the Defense			Tactical Principle of Attacking the Goal		
		M (SD)	*p*	d	M (SD)	*p*	d	M (SD)	*p*	d
Sedentary PA %	TGfU (*n* = 15)	26.28 (1.19)	<0.001	8.81	36.39 (0.85)	0.824	0.32	29.33 (1.52)	0.670	0.61
	DI (*n* = 15)	36.55 (1.14)			36.12 (0.80)			28.39 (1.52)		
Light PA %	TGfU (*n* = 15)	30.11 (0.96)	0.001	5.03	28.19 (0.54)	0.411	1.20	32.30 (2.01)	0.936	0.11
	DI (*n* = 15)	25.38 (0.92)			27.55 (0.52)			32.54 (2.01)		
Moderate PA %	TGfU (*n* = 15)	14.35 (0.65)	0.003	4.65	11.60 (0.35)	0.736	0.49	11.95 (0.73)	0.095	2.52
	DI (*n* = 15)	11.37 (0.63)			11.77 (0.33)			13.79 (0.73)		
Vigorous PA %	TGfU (*n* = 15)	29.24 (1.29)	0.178	2.01	23.80 (0.51)	0.311	1.49	26.40 (1.73)	0.656	0.65
	DI (*n* = 15)	26.69 (1.24)			24.54 (0.48)			25.27 (1.73)		
MVPA %	TGfU (*n* = 15)	43.60 (1.25)	0.004	4.50	35.41 (0.65)	0.328	1.44	38.35 (2.03)	0.809	0.40
	DI (*n* = 15)	38.05 (1.20)			36.32 (0.61)			39.07 (2.03)		

Note. *n*: sample; %: percentage; M: average; SD: standard deviation; PA: physical activity; MVPA: moderate to vigorous physical activity; d: Cohen’s d.

**Table 5:** Game Performance Evaluation Tool analysis of the attacking player with the ball according to pedagogical the approach (Teaching Games for Understanding [TGfU] vs. Direct Instruction [DI]).

		TGfU (*n* = 15)	DI (*n* = 15)	*p*	d
		M (SD)	M (SD)		
Successful executions	Pre-test	4.40 (0.68)	4.30 (0.68)	0.919	0.14
	Post-test	6.25 (0.81)	5.12 (0.81)	0.727	1.39
	% of change within groups	42.04	12.5		
Executions unsuccessful	Pre-test	3.0 (0.79)	3.40 (0.79)	0.727	0.50
	Post-test	1.12 (0.66)	3.62 (0.66)	0.018	3.78
	% of change within groups	−62.6	12.4		

Note. *n*: sample; %: percentage; M: average; SD: standard deviation; d: Cohen’s d.

**Table 6:** Game Performance Evaluation Tool analysis of the attacking player without the ball according to the pedagogical approach (Teaching Games for Understanding [TGfU] vs. Direct Instruction [DI]).

		TGfU (*n* = 15)	DI (*n* = 15)	*p*	d
		M (SD)	M (SD)		
Successful executions DM Support/Uncheck	Pre-test	7.30 (0.85)	4.66 (0.90)	0.049	3.01
	Post-test	9.00 (0.74)	4.00 (0.74)	<0.001	6.75
	% of change within groups	23.3	−14.3		
Unsuccessful executions DM Support/Uncheck	Pre-test	0.90 (0.68)	3.33 (0.71)	0.025	3.49
	Post-test	0.25 (0.57)	4.37 (0.57)	<0.001	7.22
	% of change within groups	−72.2	32.72		
Executions with successful execution Support/Uncheck	Pre-test	6.50 (0.95)	4.44 (1.00)	0.156	2.11
	Post-test	8.87 (0.70)	4.00 (0.70)	<0.001	6.95
	% of change within groups	36.61	−9.90		
Executions without success execution Support/Uncheck	Pre-test	1.20 (0.66)	3.35 (0.70)	0.027	3.16
	Post-test	0.37 (0.58)	4.37 (0.58)	<0.001	6.89
	% of change within groups	−68.3	23.0		

Note. DM: decision-making; *n*: sample; %: percentage; M: average; SD: standard deviation; d: Cohen’s d.
